# Correcting HPV Vaccination Misinformation Online: Evaluating the *HPV Vaccination NOW* Social Media Campaign

**DOI:** 10.3390/vaccines9040352

**Published:** 2021-04-06

**Authors:** Beth Sundstrom, Kathleen B. Cartmell, Ashley A. White, Henry Well, Jennifer Young Pierce, Heather M. Brandt

**Affiliations:** 1 Department of Communication, College of Charleston, Charleston, SC 29424, USA; 2 Department of Public Health Sciences, Clemson University, Clemson, SC 29634, USA; kcartme@clemson.edu; 3 Department of Public Health Sciences, Medical University of South Carolina, Charleston, SC 29425, USA; whitashl@musc.edu; 4 South Carolina Cancer Alliance, Columbia, SC 29204, USA; henry.well@sccancer.org; 5 Mitchell Cancer Institute, University of South Alabama, Mobile, AL 36604, USA; jypierce@health.southalabama.edu; 6 St. Jude Children’s Research Hospital and Comprehensive Cancer Center, Memphis, TN 38105, USA; Heather.Brandt@STJUDE.ORG

**Keywords:** HPV vaccination, campaign, misinformation, parents, social media

## Abstract

The human papillomavirus (HPV) vaccine provides protection from six HPV-related cancers. Approximately half of South Carolina adolescents have not completed the vaccination series, representing a missed opportunity to prevent cancer. The *HPV Vaccination NOW: This is Our Moment* social media campaign is an initiative of the South Carolina Cancer Alliance (SCCA) and Hollings Cancer Center at the Medical University of South Carolina (MUSC). This statewide social media campaign aimed to increase parental awareness of and build vaccine confidence around HPV vaccination in S.C. The ten-week campaign was strategically implemented between June and August 2019 to encourage HPV vaccination at back-to-school medical appointments. A process evaluation showed that the campaign resulted in over 370,000 total impressions, reached over 33,000 individuals, and culminated with over 1122 followers. There were over 2700 engagements on Facebook and Twitter. A qualitative content analysis indicated that pro-vaccine and anti-vaccine comments were dominated by personal stories. Comments promoting misinformation about the HPV vaccine were often countered through peer-to-peer dialogue. Findings suggest that creating opportunities for the target audience to engage with campaign messages effectively corrected misinformation.

## 1. Introduction

The United States (U.S.) could eliminate cervical cancer by 2059 through increasing human papillomavirus (HPV) vaccination and improving participation in cervical cancer screening and treatment [1]. Several studies show declining rates of HPV infection and cervical precancers in the U.S. since the introduction of the HPV vaccine [2]. The HPV vaccine provides protection from six HPV-related cancers, including cervical, vaginal, and vulvar cancer, oropharyngeal cancer, penile cancer, and anal cancer. Research shows the importance of a nonavalent vaccine to improve protection against HPV infections [3]. As of 2020, Gardasil^®^ 9, a nonavalent vaccine, is the only HPV vaccine available in the United States. In the U.S., HPV causes approximately 35,900 of the 45,300 new cases of HPV-associated cancer among men and women each year [4]. A recent study in Sweden found that HPV vaccination was associated with a significantly reduced risk of invasive cervical cancer [5]. In 2020, the World Health Organization (WHO) launched a global movement to eliminate cervical cancer by increasing HPV vaccination, screening, and treatment [6]. Although cervical dysplasia after HPV vaccination is rare [7], efforts to eradicate cervical cancer rely on increasing vaccination, screening, and treatment.

Despite having been available since 2006 in the U.S., uptake of the HPV vaccine remains lower than other adolescent vaccines. In the U.S., HPV vaccination rates are below those of other adolescent vaccines available, and rates remain lower than in other developed nations [8]. Australia, for example, has achieved 78.6% vaccination completion coverage rates among girls and 72.9% among boys turning 15 years old [9]. According to the 2019 NIS-TEEN vaccination data that reports on HPV vaccination rates among adolescents in the U.S., 71.5% of all adolescents have received one or more doses and 54.2% of adolescent girls and boys are up-to-date on the HPV vaccine [10]. In a recent study among unvaccinated adolescents, 58% of parents indicated no intention to start the HPV vaccine series, revealing a significant public health issue [11].

Almost half of South Carolina adolescents have not completed the vaccination series, representing a missed opportunity to prevent cancer. In 2016, South Carolina had the lowest rate of HPV vaccine completion among adolescent girls in the U.S. (30.8%) and the second lowest rate of up-to-date vaccination among adolescent boys (27.4%). Although South Carolina has made significant improvements in HPV vaccination initiation (71.8%) and completion (53.0%), completion rates remain slightly below the U.S. average (54.2%) [10]. Data show that the COVID-19 pandemic has resulted in substantially lower uptake of adolescent vaccinations, especially HPV, and scholars suggest urgent interventions are necessary to avoid losing any and all gains [12]. A recent study showed a 71% drop in healthcare visits among 7–17 year olds in the U.S. [13]. An analysis of immunizations provided by the Vaccines for Children Program in 2020–2021 compared to 2019 showed that adolescent immunization dropped by approximately 21% or more than 1 million doses since the start of the pandemic [14]. In lieu of access to in-person interactions with a health care provider, other sources of health-related information, including social media, may become increasingly important [15].

Social media remains a promising, although underutilized, tool in HPV vaccination promotion with limited evidence evaluating extant interventions [16,17]. Experts have identified how to increase HPV vaccine confidence through social media interventions as a research priority [17]. Maher and colleagues [18] reviewed 10 studies that used social media as a platform for implementing health behavior interventions and found that nine of the studies reported significant improvements in health behavior outcomes related to behavior change. A recent analysis of a Danish social media campaign found that a heart-brain communication strategy, which combined facts and emotions successfully addressed HPV vaccine hesitancy [19]. This campaign also found that personal stories supported by factual information effectively promoted positive dialogue on Facebook and supported informed decision making among HPV vaccine hesitant mothers [20]. In the U.S., approximately 60% of adults look online for information about health [21]. The majority of Americans report having used Facebook (69%) with almost one quarter of Americans active on Twitter (22%). Seventy-five percent of women use Facebook compared to 63% of men, and 79% of people aged 18–49 report using Facebook [22]. Social media, therefore, can be used to reach a significant percent of the U.S. adult population when implementing public health interventions.

In addition to serving as a primary source of health information, scholars argue social media may provide an effective platform to correct misinformation and address myths about vaccination [23]. Some studies have shown that exposure to negative anti-vaccine posts on social media may lead to lower vaccination rates [24]. According to a report by the Center for Countering Digital Hate (CCDH), anti-vaccine social media accounts have increased their followers by more than 7.8 million people since 2019, with 31 million people following anti-vaccine groups on Facebook [25]. A recent study found that four out of ten Facebook posts about HPV vaccination included messages that magnified the risk of the vaccine [26]. Another study of HPV vaccine misinformation on social media found that anti-vaccine posts were more likely to include personal narratives and to focus on concealment, injury, and conspiracy theories [27]. Approximately 19% of parents reported hearing stories about HPV vaccine harms in social and traditional media and these parents were less likely to initiate HPV vaccination compared with parents who did not hear any stories [28]. Experts have called for research about how health (mis)information spreads and what public health strategies might help control the spread of health misinformation and disseminate correct information [29].

The purpose of this study was to investigate how the *HPV Vaccination NOW* social media campaign addressed HPV vaccination misinformation online and fostered message co-creation with the target audience. Formative audience research and a pilot campaign guided the development and implementation of the social media campaign. The campaign aimed to raise the voices and stories of parents in South Carolina. Messaging focused on compassion and understanding, recognizing that all parents want to do what is best for their children and families. This approach was designed to empower parents and appealed to their moral responsibility and personal choice to protect children from cancer. The research study was designed to answer two research questions: (1) What myths and misinformation about HPV vaccination emerged through the *HPV Vaccination NOW* social media campaign? and (2) How did the campaign correct misinformation to help mitigate its impact?

## 2. Materials and Methods

The ten-week campaign was strategically implemented between 5 June and 10 August, 2019 to encourage HPV vaccination at back-to-school medical appointments. Two Social media platforms, Facebook and Twitter, were selected to target parents in South Carolina by providing information and addressing misinformation. A new Medical University of South Carolina (MUSC) website was created to address misinformation and offered information for making an appointment to get the HPV vaccine at MUSC, through a primary care provider, or at a Department of Health public health clinic (https://muschealth.org/hpv (accessed on 1 January 2021). The campaign’s call-to-action included prompts to “Like”/“Follow” social media platforms. Researchers employed a mixed methods approach to evaluation, including (1) a campaign process evaluation, including message split-testing and social media metrics and (2) a qualitative content analysis of user-generated content.

### 2.1. HPV Vaccination NOW

**Figure 1:**
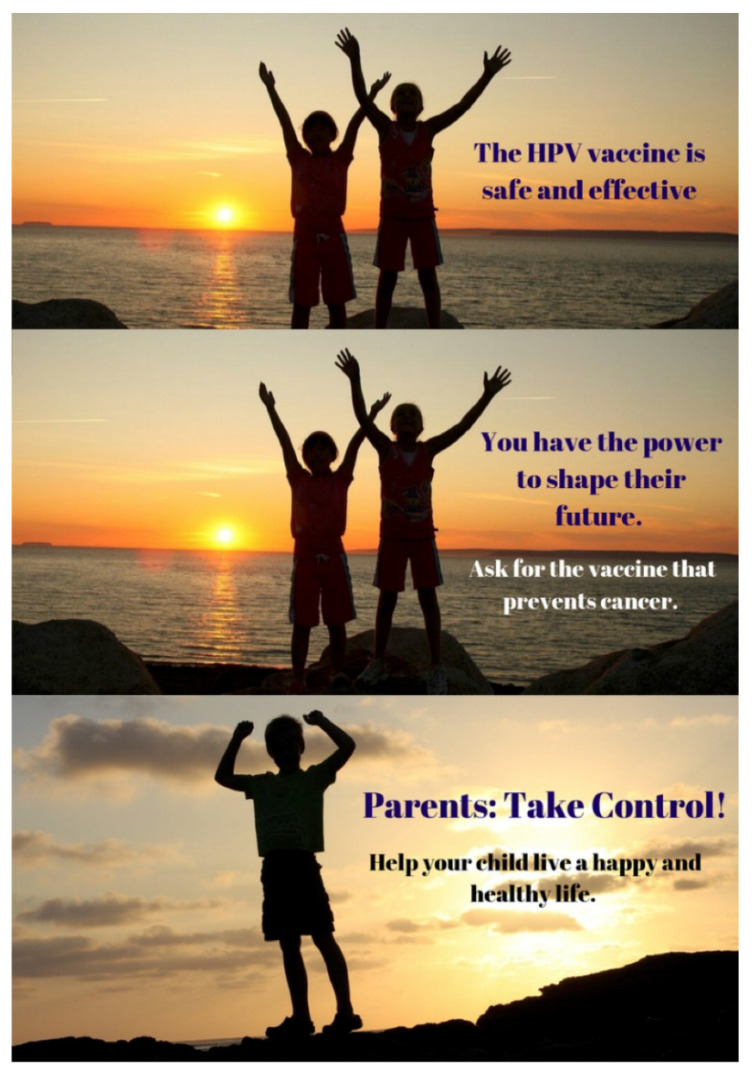
Top three performing ads.

The *HPV Vaccination NOW* social media campaign is an initiative of the state comprehensive cancer control coalition, South Carolina Cancer Alliance (SCCA), and the National Cancer Institute-designated Hollings Cancer Center at MUSC based in Charleston, South Carolina. The *HPV Vaccination NOW* campaign tagline was “*This is Our Moment*,” and the slogan was “*South Carolina is gaining momentum. We have made significant improvements in HPV vaccination rates. This is our moment. #HPVvaxNOW #OurMoment.*” See Figure 1 for the top three performing ads. This statewide social media campaign aimed to increase parental awareness of and build vaccine confidence toward HPV vaccination in South Carolina. The goal of this campaign was to increase HPV vaccination in the state through consistent messaging that the HPV vaccine is essential for protecting the health of our children. The target audience included parents of pre-teens and teenagers, which included women and men between the approximate ages of 30–50 living in South Carolina. Advertisements were served to the target audience based on age, location, and parent demographic characteristics. Formative audience research and a pilot campaign evaluation [30] guided the development and implementation of the social media campaign. A content analysis of online HPV vaccination in South Carolina identified two primary misconceptions about the HPV vaccination: concerns about safety and that the vaccine could increase sexual activity among adolescents [31]. Qualitative interviews with experts in South Carolina identified three elements of effective messages: (1) appropriate trusted messengers, (2) factual messages about HPV and the vaccine with a focus on overcoming misunderstanding about the vaccine’s purpose and target groups, and (3) strategic communication to ensure effectiveness and reach of messages to the intended audience [32].

Formative audience research led to the insight that parents in South Carolina are compassionate, caring, and thoughtful. They want to do what is best for their children and their families. As a result, *HPV Vaccination NOW* aimed to raise the voices and stories of parents in the state as primary, trusted messengers. Messages focused on compassion and the understanding that all parents want to do what is best for their children and families. The campaign also focused on empowerment and appealing to parents’ moral responsibility and personal choice to protect their children against cancer. The campaign capitalized on progress and momentum by focusing on South Carolina’s significant improvement in vaccination rates. In line with national campaigns, *HPV Vaccination NOW* emphasized cancer prevention, normalizing HPV vaccination as a routine part of the vaccination series, and the safety, effectiveness, and long-lasting protection of the HPV vaccination. A social media editorial calendar was created with 3–5 posts per day on Facebook and Twitter with a different theme each week (see Table 1). A partner toolkit was developed which included one post per day, and it was disseminated to likeminded organizations who shared the posts with their networks to amplify the campaign’s messages.

### 2.2. Campaign Evaluation

#### 2.2.1. Process Evaluation

Process evaluation measured the implementation of the campaign, including reach and frequency of message exposure or dose [33]. Researchers measured key performance indicators (KPIs), including exposure, reach, and engagement. Reach was measured through impressions, including the number of times the ads were displayed, as well as the number of individuals who saw the ads. Individual social media platforms were used to collect analytics data. Researchers documented engagement with specific messages and ads by measuring impressions, views, clicks, likes, comments, shares, and retweets. In addition, researchers tracked the click-through-rate (CTR), which measures the number of individuals clicking on a link through the ads. Researchers tested the same messages paired with either a photograph or an abstract graphic using split testing. Split-testing divides the audience into random, non-overlapping groups and tracks the performance (or cost) of each ad to reach the target audience given uniform spending across all ads. This randomization helps ensure the test is conducted fairly because other factors do not skew the results of the group comparison. The split-test also determines the confidence level, which is a number that represents how likely it is that the campaign would get the same results if the test was run again. The ad sets tested included the same message with only one meaningful difference, either a graphic OR a photograph/image. All ads included the following body text:

“*HPV Vaccination NOW: This is Our Moment* is a new initiative by @sccancerorg and @MUSCHollings. The HPV vaccine protects girls and boys from 6 HPV-related cancers. Make an appointment to get your girls and boys the HPV vaccine today. #HPVvaxNOW #OurMoment http://muschealth.org/hpv” (accessed on 1 January 1 2021)

#### 2.2.2. Qualitative Content Analysis

A qualitative content analysis was performed to analyze public posts on the *HPV Vaccination NOW* social media platforms. Content analysis is a well-established method to investigate vaccine misinformation on social media [34,35]. All posts were combined for data analysis. A constant comparative method was used to analyze these data. HyperRESEARCH 4.5.0 qualitative data analysis software was used for data analysis. The first author coded posts line-by-line, allowing themes and patterns to emerge from these data. To ensure credibility, the research team met to discuss emerging patterns and concepts in order to develop a codebook. Next, axial coding identified cross-cutting patterns [36]. To ensure reliability, researchers employed analytic tools, such as asking practical, theoretical, and sensitizing questions to develop provisional answers and to think outside of the box [36]. Making comparisons at the property and dimensional level of constructs advanced the data analysis from description to abstraction [36]. The research team met to discuss and resolve issues with coding, ensure consistency, and achieve unanimous consensus. Researchers agreed on patterns, themes, and implications of the data through discussion.

## 3. Results

The primary target audience of the *HPV Vaccination NOW* social media campaign included parents of pre-teens and teenagers, specifically women and men between the ages of 30 and 50 across the state of South Carolina. Since social media allows anonymity, identifying the exact demographic breakdown of the target audience was not possible. The campaign’s followers with public demographic information suggested that the campaign reached the intended target audience. The majority of followers on Facebook were between the ages of 25 and 44 years. The gender breakdown was 88% women and 12% men. On Twitter, the gender breakdown was 61% women and 39% men. Individuals interacted with the campaign from 30 out of 46 counties in South Carolina, or at least 65% of the state. There was robust engagement across each region of South Carolina, including the Upstate, Midlands, Pee Dee, and the Lowcountry regions.

### 3.1. Process Evaluation

A process evaluation indicated that the *HPV Vaccination NOW* campaign resulted in significant exposure and strong saturation of the campaign messages (See Table 2). Researchers determined saturation based on impressions, reach, and engagement. Reach shows the total number of unique individuals who saw content from HPV vaccination NOW in their Facebook news feed over a 7-day period. The campaign avoided message fatigue by monitoring engagement in relation to reach. Split-testing revealed a preference for messages paired with a photograph, instead of a graphic (See Table 3).

The campaign resulted in over 370,000 total impressions, reached over 33,000 individuals, and culminated with over 1122 followers. There were over 2700 engagements on social media (see Table 4). Facebook and Twitter offered an excellent return on investment with a cost-per-click (CPC) rate of USD 1.13 and USD 1.04, respectively. The national cost-per-click average in the health care industry is USD 1.32.

### 3.2. Content Analysis

The content analysis revealed myths and misinformation about HPV vaccination that emerged through the *HPV Vaccination NOW* social media campaign and how the campaign corrected misinformation to help mitigate its impact. The content analysis also revealed strategies that were most effective to engage the target audience. The top social media posts uniformly engaged trends and current conversations. For example, the social media post that received the most engagement was a part of the #FF hashtag conversation, “It is Follow Friday #FF! Each week, we will be highlighting HPV vaccination successes around the world.” Other conversation starters included Woman Crush Wednesday #WCW and Thankful Thursday #TT. The content analysis revealed myths and misinformation about the HPV vaccination, including concerns about safety and side effects, effectiveness, and sexual activity. Two primary themes emerged related to how the campaign corrected misinformation to help mitigate its impact, including (1) addressing misinformation and (2) stimulating peer-to-peer dialogue.

### 3.3. Addressing Misinformation

Pro-vaccine and anti-vaccine comments were dominated by personal stories. Pro-vaccine comments often mentioned the importance of vaccinating sons and daughters based on personal experiences with cancer. According to one parent:

“Got my son and daughter vaccinated against HPV. I know too many women diagnosed with cervical cancer or pre-cancerous conditions not to want to protect my own kids from this virus. Vaccination rates are much higher in other developed countries while Americans continue to deny the benefits of life saving vaccines to their children over false information and anti-science viewpoints.”

Anti-vaccine comments frequently described personal experiences with vaccinations. One parent said that HPV vaccination was the:

“Worst thing I ever did for my daughter. Made her gain 60 pounds and caused what looks like permanent body aches and has so much trouble sleeping since then. They are terrible, I would never recommend them. Never.”

The *HPV Vaccination NOW* campaign did not delete comments such as this one. Researchers deleted and/or blocked spam, hate speech, inappropriate/offensive language, personal attacks, and trolls (i.e., individuals who purposefully attack others online through disruptive posts). All other comments were allowed and researchers replied to all posts, while also providing an opportunity for the target audience to engage with the campaign messages.

The most common posts featuring misinformation about the HPV vaccination described concerns about safety and side effects, effectiveness, and sexual activity. Misinformation related to safety often focused on the HPV vaccination as new and untested. According to one parent, “Because it isn’t safe at all…They haven’t had enough clinical trials to make sure that it’s safe. Everything is for profit with side effects being overlooked.” Many comments opposed the HPV vaccination because it prevented transmission of a sexually transmitted infection (STI). According to one post, “How about teach kids about safe sex not vaccines to prevent std?” Researchers replied to all comments with empathy and provided credible information from third-party sources, such as the Centers for Disease Control and Prevention (CDC). Responses started with an acknowledgment that researchers welcomed engagement with the campaign and reinforced our core value of compassion. For example, many posts started with, “We appreciate your comment. We believe that all parents in South Carolina want to do what is best for their children and families.” If a parent shared a negative vaccine experience, the response began, “We appreciate hearing from you and understand your son/daughter had a negative experience.” Table 5 includes standard responses to the most frequent misinformation topics encountered during the campaign.

### 3.4. Stimulating Peer-to-Peer Dialogue

Comments promoting misinformation about the HPV vaccine were often countered through peer-to-peer dialogue. Campaign followers on Facebook and Twitter often provided direct rebuttals to misinformation. When one parent shared a negative vaccine experience, “my son almost fainted, couldn’t raise his arm for two days and slept for about ten hours straight. Won’t be getting it again,” another parent posted, “sure beats brain and throat cancer though.” In another exchange, when one parent described an inaccurate side effect, “it causes early infertility too!” another parent countered with “cervical cancer leads to infertility.” In another discussion, one parent linked the HPV vaccine to sexual activity, “my children won’t be getting it. Unless you have sex, you can’t get the disease. And my children are raised to wait until marriage. Shame on y’all promoting this junk.” Another parent responded, “HILARIOUS…so…because they wait until marriage…they can’t get HPV??? UUUMMM WRONG ANSWER! You don’t have to be a child!! This is not promoting children having sex. This vaccine is to prevent HPV related cancers. Vaccines = Prevent.”

Many campaign followers engaged in peer-to-peer dialogue by sharing their reasons for vaccinating their children. In response to parents who posted about adverse vaccine events, many parents posted positive experiences. According to one parent, “Had both my daughters vaccinated and there were no issues!” Other parents described the HPV vaccine as an opportunity to protect their children and keep them safe. According to one parent, “Made sure my daughter got this vaccine. It’s another step I was able to take to ensure her safety.” Parents reinforced campaign messages by sharing their own stories. According to one parent, “I had my youngest son and daughter vaccinated. It is important.”

## 4. Discussion

The results from the process evaluation indicate that the *HPV Vaccination NOW: This is Our Moment* campaign demonstrated significant exposure and strong saturation of the campaign messages. In only ten weeks, the campaign resulted in over 370,000 total impressions, reached over 33,000 individuals, and culminated with over 1122 followers. There were over 2700 engagements on social media. In addition, Facebook and Twitter offered an excellent return on investment with a cost-per-click (CPC) rate below the national average. Reach decreased in weeks 7 and 8, while beginning to increase again in weeks 9 and 10. It is likely that these numbers reflect real-time campaign adjustments designed to avoid message fatigue and audience burn-out. Furthermore, these results were achieved with a total advertising budget of approximately USD 2000. The *HPV Vaccination NOW* social media campaign provides a cost-effective model for comprehensive cancer control coalitions and other non-profit organizations to make a minimal investment for a substantial return on investment. This approach could be scaled to maximize impact on HPV vaccination and build resilience against anti-vaccination myths and misinformation.

The *HPV Vaccination NOW* social media campaign offers best practices for effectively responding to HPV vaccination misinformation in order to limit the impact of false information online. The content analysis revealed strategies that were most effective to engage the target audience. The top social media posts uniformly engaged trends and current conversations, including Follow Friday #FF, Woman Crush Wednesday #WCW and Thankful Thursday #TT. This finding highlights the importance of providing content that offers value to the target audience in order to increase engagement. Split-testing revealed a preference for messages paired with a photograph, instead of a graphic. In line with current research, a photograph may increase the emotional value of the advertisement and resonate more strongly with parents [20].

The content analysis also identified myths and misinformation about HPV vaccination that emerged through the *HPV Vaccination NOW* social media campaign and how the campaign corrected misinformation to help mitigate its impact. The most common posts featuring misinformation about the HPV vaccination described concerns about safety and side effects, effectiveness, and sexual activity. In particular, safety concerns were often based on the vaccine as “new and untested” with some posts expressing a lack of trust for pharmaceutical companies and the government because the vaccine was created “for profit with side effects being overlooked.” These sentiments reflected recent research that found antivaccine messages on Twitter focused on safety concerns and conspiracy theories [35]. As a result, campaign planners should continue to provide up-to-date vaccine safety information in plain language.

Findings revealed that pro-vaccine and anti-vaccine comments were dominated by personal stories. These narratives often described health impacts of the vaccine or HPV-related cancer. In line with recent research calling for campaigns to include stories of HPV vaccine preventable diseases [28], results suggest the importance of emphasizing HPV vaccination as cancer prevention. In addition, posts described misinformation about the potential health consequences of the vaccine, including infertility. This finding builds on a recent study, which showed anti-vaccine posts were more likely to include personal narratives and to focus on concealment, injury, and conspiracy theories [27]. Campaign planners should be prepared to address concerns about infertility as well. This study also shows that these comments are often presented as personal stories or narratives, which may be a more effective way to address misinformation.

The *HPV Vaccination NOW* social media campaign created opportunities for the target audience to engage with campaign messages. The campaign created a welcoming space for parents to discuss the HPV vaccine by focusing on compassion and understanding, recognizing that all parents want to do what is best for their children and families. Researchers ensured a safe space for discussion by responding to and engaging with all posts. However, researchers avoided responding to individual comments, choosing to provide responses at the post-level provided an opportunity to correct misinformation among readers without amplifying anti-vaccine comments. This finding extends recent research on the importance of developing a community management strategy to facilitate dialogue about HPV vaccination [19]. By encouraging dialogue, the *HPV Vaccination NOW* campaign fostered message co-creation with the target audience. Peer-to-peer dialogue transformed the campaign into a space for parents to interact with one another, which effectively corrected misinformation. By empowering parents to share accurate information and supporting parents as spokespeople in support of HPV vaccination, the campaign leveraged peer-to-peer dialogue to mitigate the spread of misinformation.

## 5. Conclusions

This study contributed to the call by scholars to better understand public health strategies to address misinformation and disseminate accurate information on social media [29]. After learning about the *HPV Vaccination NOW* social media campaign through a series of invited expert panels, the U.S. Department of Health and Human Services’ (HHS) Office on Women’s Health (OWH) launched the *HPV VAX NOW* campaign to increase HPV vaccination rates among young adults ages 18–26 in Mississippi, South Carolina, and Texas in January 2021. Despite the strengths and success of the campaign, the results are limited by the real-world conditions in which this study was conducted. A qualitative content analysis highlights robust qualitative data in the form of quotes embedded and contextualized in the results, which contributes to the credibility and transparency of qualitative research [36]. However, one of the limitations of qualitative research is that the findings are not necessarily generalizable beyond this case. Future studies may seek to incorporate additional cases and/or employ an experimental research design. In addition, future research may investigate how to communicate about recent findings suggesting the promise of HPV vaccination among older adults and the potential for therapeutic vaccination as an adjuvant approach among patients being treated for cervical cancer [37,38]. Despite these limitations, this study provides an exploratory analysis of a successful statewide HPV vaccination social media campaign. This study identified myths and misinformation about HPV vaccination that emerged through the *HPV vaccination NOW* social media campaign, as well as how campaign strategies mitigated the spread of misinformation.

## Figures and Tables

**Table 1:** Editorial calendar with sample social media posts.

Theme	Sample Post
**Week 1 (June 5–8)**	
Introduction to *HPV Vaccination NOW* Social Media Campaign	About 14 million people, including teens, become infected with HPV each year. HPV infection can cause cervical, vaginal, and vulvar cancers in women and penile cancer in men. HPV can also cause anal cancer, throat cancer, and genital warts in both men and women. The good news is that you can get vaccinated against HPV and prevent cancer. Vaccines work! #2shots2stopCancer #HPVvaxNOW https://www.cdc.gov/hpv/parents/cancer.html (accessed on 1 January 2021)
**Week 2 (June 9–15)**	
About HPV Vaccination	Did you know? The HPV vaccine is for girls AND boys, ages 9–26. The Advisory Committee on Immunization Practices (ACIP) recommends a 2-dose schedule (0, 6–12 months) for HPV vaccination of girls and boys who initiate the vaccination series at ages 9 through 14 years. #2shots2stopCancer #HPVvaxNOW #OurMoment https://www.cdc.gov/hpv/parents/vaccine.html (accessed on 1 January 2021)
**Week 3 (June 16–22)**	
Focus on Family/Parents	As parents, you do everything you can to protect your children’s health for now and for the future. Today, there is a strong weapon to prevent several types of cancer in our kids: The HPV vaccine. #HPVvaxNOW #OurMoment https://www.cdc.gov/vaccines/parents/diseases/teen/hpv-indepth-color.pdf (accessed on 1 January 2021)
**Week 4 (June 23–29)**	
Focus on Clinicians	Clinicians: Providing personal examples of how you support vaccinations for your family members shows you believe they are important. Share how you recommended or administered HPV vaccine for your own children, grandchildren, nieces, or nephews. Sharing your personal experience may make parents more comfortable in their decision to vaccinate their child. #HPVvaxNOW #OurMoment https://www.cdc.gov/hpv/hcp/boosting-vacc-rates.html (accessed on 1 January 2021)
**Week 5 (June 30–July 6)**	
HPV Vaccination in South Carolina	Most parents choose the HPV vaccine for their children. In South Carolina, almost 60% of teens receive the first dose. To protect the next generation through herd immunity, we need to reach 80% of young adults. The HPV vaccination can protect a generation of South Carolina preteens and teens from cancers caused by HPV. #HPVvaxNOW #OurMoment https://www.cdc.gov/mmwr/volumes/67/wr/mm6733a1.htm (accessed on 1 January 2021)
**Week 6 (July 7–13)**	
Focus on Cervical Cancer	Eliminating cervical cancer depends on successful HPV vaccination and cervical screening initiatives. According to Professor Karen Canfell from the Cancer Council New South Wales, “We must be very clear about this: millions of women can be spared unnecessary, terrible suffering if HPV vaccines can be effectively deployed and scaled up globally.” https://www.theguardian.com/society/2019/feb/20/cervical-cancer-hpv-vaccine-screening-research?fbclid=IwAR30FdW4q8bgIM1k27ByhelsLb2lX9FS7DibQ2ZbopzhSLWBdjF3Bsno0Ec (accessed on 1 January 2021)
**Week 7 (July 14–20)**	
Focus on Head, Neck, and Throat cancers	Did you know about the link between HPV and head and neck cancers? Men are twice as likely to get head and neck cancers compared with women, but new cases of head and neck cancers in women are increasing. #HPVvaxNOW https://www.cdc.gov/cancer/headneck/ (accessed on January 1, 2021)
**Week 8 (July 21–27)**	
Focus on Survivor Stories	Check out these Cervivor Stories where survivors tell their stories to challenge the stigma of cervical cancer and build a movement to end cervical cancer. There is strength in stories. https://cervivor.org/stories/ (accessed on January 1, 2021)
**Week 9 (July 28-Aug 3)**	
Focus on Facts about HPV and Answering Tough Questions	The HPV vaccine protects fertility by preventing pre-cancerous cervical lesions and cervical cancer. Treatment of pre-cancerous lesions can sometimes lead to pregnancy loss, preterm delivery, low birthweight, or other complications in the future. In the United States alone, approximately 400,000 women present with abnormal Pap test results each year. Reducing pre-cancerous cells means that fewer women will have to experience the psychological and physical impact of treatment. #HPVvaxNOW #OurMoment http://www.euro.who.int/__data/assets/pdf_file/0010/356842/QA_HPV_General_EN.pdf?ua=1 (accessed on 1 January 2021)
**Week 10 (Aug. 4–10)**	
Focus on Activism (What you can do)	Social media makes a difference! A recent study in Pediatrics found that pregnant women who interacted with responsible vaccine information on social media were more likely to vaccinate their children on time. We all play an important role in increasing vaccine understanding and acceptance.

**Table 2:** Digital media performance by channel.

	Impressions	Reach	Engagement
**Week 1 (June 5–8)**			
Facebook	15,116	8590	146
Twitter	12,336	N/A	110
**Week 2 (June 9–15)**			
Facebook	25,413	11,755	221
Twitter	17,595	N/A	135
**Week 3 (June 16–22)**			
Facebook	23,467	11,696	278
Twitter	14,436	N/A	126
**Week 4 (June 23–29)**			
Facebook	23,682	11,108	85
Twitter	12,330	N/A	109
**Week 5 (June 30–July 6)**			
Facebook	25,799	11,792	111
Twitter	18,392	N/A	162
**Week 6 (July 7–13)**			
Facebook	14,263	9288	78
Twitter	18,219	N/A	174
**Week 7 (July 14–20)**			
Facebook	12,835	5359	91
Twitter	20,542	N/A	130
**Week 8 (July 21–27)**			
Facebook	17,001	5876	178
Twitter	20,313	N/A	125
**Week 9 (July 28–Aug 3)**			
Facebook	16,974	6248	102
Twitter	20,432	N/A	133
**Week 10 (Aug. 4–10)**			
Facebook	15,211	6118	91
Twitter	20,364	N/A	155

**Table 3:** Top performing ads based on split-testing.

Ad Name	Text	Cost Per Result	Reach	Impressions	Spend
Parents Take Control (Graphic)	Parents: Take Control! Help your child live a happy and healthy life.	USD 6.28	6374	11.2 K	USD 40
* + Parents Take Control (Image)		USD 5.98	6684	12.2 K	USD 40
Shape Their Future (Graphic)	You have the power to shape their future. Ask for the vaccine that prevents cancer.	USD 6.43	6221	10.5 K	USD 40
* + Shape Their Future (Image)		USD 6.17	6486	11.5 K	USD 40
Safe and Effective (Graphic)	The HPV vaccine is safe and effective.	USD 5.73	6978	12.7 K	USD 40
* ^ Safe and Effective (Image)		USD 5.69	7024	13.2 K	USD 40

* The winning ad provided the lowest cost per result (in this case, cost per 1000 People Reached). + There’s a 90% chance or greater of the same winner if the test ran again. ^ There’s a 66% chance of the same winner if the test ran again.

**Table 4:** Campaign dose key performance indicators.

	Impressions	Reach	Engagement	Clicks (Paid)	CTR * %	Cost-Per-Click USD	Followers	Spend USD
**Overall**								
Facebook	188,196	33,951	1362	797	0.44	1.13	515	900.00
Twitter	182,700	N/A	1365	984	0.71	1.04	597	1019.69

* CTR = Click-through-rate.

**Table 5:** Standard responses to misinformation posts.

Topic	Response *
Safety	The safety of HPV vaccination was tested in thousands of volunteers before the vaccines were approved. Over the last decade, more than 100 million doses have been distributed in the United States. The HPV vaccine has been carefully studied and has been shown to be safe, effective, and long-lasting.
Side effects	The most common side effects after HPV vaccine are mild and include pain in the arm where the shot was given, fever, dizziness, and nausea. Approximately 100 million doses of HPV vaccine have been distributed in the U.S. since the vaccine was introduced, and no serious side effects have been linked to HPV vaccination.
Effectiveness: Does the vaccine work?	Ongoing studies show that the HPV vaccination works very well. Since becoming available in 2006, this vaccine has already decreased HPV infection, genital warts, and precancers in young people.
Effectiveness: Risk	HPV is so common that almost everyone will be infected at some point in their lives. Most parents in South Carolina choose the HPV vaccine for their children. Almost 60% of teens in our state receive the first dose. The HPV vaccine prevents six cancers and other diseases in both girls and boys.
Infertility	Clinical trials before the first HPV vaccine was licensed in 2006 and safety monitoring and studies since its introduction have confirmed that the vaccine does not cause any reproductive problems in women.
Sexual activity: Promotion	Studies show that HPV vaccination does not lead to increased sexual activity or sex at a younger age. Getting the HPV vaccine before your child is exposed to the virus can prevent 6 HPV-related cancers. Even someone who waits until marriage for sex and only has one partner can still get HPV. The HPV vaccine is recommended for boys and girls when they are 11 to 12 years old. The HPV vaccine is more effective when given at this age rather than waiting until a child is older.
Sexual activity: Wait until marriage	We know that all parents in South Carolina want to do what is best for their children and their families. HPV is so common that almost everyone will be infected at some point in their lives. Anyone who is sexually active can get HPV, even if you have had sex with only one person. The HPV vaccine prevents 6 HPV-related cancers in girls and boys.
Sexual activity: Condom	Using condoms the right way every time you have sex can lower your chances of getting HPV. But HPV can infect areas not covered by a condom—So condoms may not fully protect against getting HPV. The HPV vaccine prevents 6 HPV-related cancers in girls and boys.

* All responses started with: “Thank you for commenting! We appreciate hearing from you.” Responses also included a link to a credible third-party source of information, such as the Centers for Disease Control and Prevention (CDC).
